# Implementation of a digital distress detection system in palliative care: qualitative data on perspectives of a multiprofessional palliative care team

**DOI:** 10.1186/s12904-024-01530-3

**Published:** 2024-08-07

**Authors:** Katharina Seibel, Claudia Lorena Orellana Rios, Titus Sparna, Carola Becker, Jan Gaertner, Gerhild Becker, Christopher Boehlke

**Affiliations:** 1 Department of Palliative Medicine, Faculty of Medicine, University Medical Center Freiburg, University of Freiburg, Freiburg, Germany; 2 Current address: Helios Klinikum Bonn/Rhein-Sieg, Bonn, Germany; 3 Current address: Department of Clinical Research, University Hospital Basel, University of Basel, Basel, Switzerland; 4 Palliative Care Center Basel, Basel, Switzerland

**Keywords:** Palliative care, Specialist palliative care, Distress, Sensor system, Healthcare workers, Digital health technologies, Qualitative research

## Abstract

**Background:**

Digital health technologies such as sensor systems are intended to support healthcare staff in providing adequate patient care. In the Department of Palliative Medicine (University Medical Center Freiburg), we developed and implemented a noninvasive, bed-based sensor system in a pilot study. The aim was to detect distress in patients who were no longer able to express themselves by monitoring heart and respiratory rates, vocalizations, and movement measurements. The sensor system was intended to supplement standard care, which generally cannot guarantee constant monitoring. As there is a lack of data on how healthcare professionals experience such a techno-digital innovation, the aim of this study was to explore how the multiprofessional palliative care team who piloted the sensor system perceived its potential benefits and limitations, and how they experienced the broader context of healthcare technology and research in palliative care.

**Methods:**

We conducted a qualitative interview study with 20 members of the palliative care team and analyzed the recorded, verbatim transcribed interviews using qualitative content analysis.

**Results:**

The sensor system was described as easy to use and as helpful support for patients, care staff, and relatives, especially against the backdrop of demographic change. However, it could not replace human interpretation of stress and subsequent treatment decisions: this remained the expertise of the nursing staff. A potential reduction in personnel was expected to be a risk of a digital monitoring system. The special conditions of research and digital health technologies in an end-of-life context also became clear. Specifically, healthcare staff were open to health technologies if they benefited the patient and were compatible with professional nursing and/or palliative care attitudes. Additionally, a patient-protective attitude and possible interprofessional differences in priorities and the resulting challenges for the team became apparent.

**Conclusions:**

A potential digital solution for distress monitoring was considered useful by palliative care practitioners. However, interprofessional differences and compatibility with existing palliative care practices need to be considered before implementing such a system. To increase user acceptability, the perspectives of healthcare professionals should be included in the implementation of technological innovations in palliative care.

**Supplementary Information:**

The online version contains supplementary material available at 10.1186/s12904-024-01530-3.

## Background

Due to the known sociodemographic changes and the increasing prevalence of multimorbidity, the need for palliative and end-of-life care is expected to increase significantly [1–3]. This goes hand in hand with the challenge of current and increasing staff shortages in the healthcare sector, which will also have an impact on palliative care [4].

Digital health technologies, defined as “the use of information and communications technologies in medicine and other health professions to manage illnesses and health risks and to promote wellness“ [5], can provide assistance here. They support healthcare staff in day-to-day tasks, reducing their workload [6], and are therefore seen as a contribution to mitigating the negative effects of demographic change on society [7]. Examples from the field of cancer and palliative care include remote symptom monitoring during chemotherapy [8] and video consultations in specialized palliative care at home [9].

Sensor systems are also classified here as a means of supporting good patient care, e.g. through monitoring or preclinical diagnostic support. As mobile or embedded systems, they are often continuously automated and work without human interaction—for example, to measure physical activity or to detect medical emergencies [10]. In recent years, therefore, there has been increasing research into contact-free sensor systems that enable the measurement of vital signs and even the pathophysiological changes associated with dying [11–16].

In a pilot study, the Department of Palliative Medicine at the University Medical Center Freiburg and partners developed a contactless sensor system to detect distress in patients who were no longer able to express themselves, as is the case in advanced neurogenerative diseases such as dementia or advanced cancer with cognitive impairment [17, 18]. Distress in palliative care encompasses emotions ranging from sadness to more complex psychological syndromes such as depression and anxiety disorders [19]. The term distress, as used here, can be caused by somatic symptoms like pain, dyspnea, or agitation, which can lead to psychological suffering. It emphasizes the interdependence of physical and psychological symptoms, where one can trigger the other [20] and affect quality of life [21]. Currently, it is still challenging for formal and informal caregivers to continuously monitor distress in patients being unable to communicate, e.g. via changes in motor activity (restlessness, hypoactivity or signs of relieving postures), by paying attention to crying, moaning or other “verbal” indicators of discomfort, and by recognizing changes in the activity of the autonomic nervous system (e.g. tachycardia, tachypnoea) as well as facial expressions [22, 23]. Therefore, in addition to the current gold standard, which is based on observer-rated distress [24–26], the aim was to develop a tool to assist nurses in monitoring distress, as the constant, round-the-clock bedside monitoring of patients by caregivers and staff is almost impossible to provide in most care settings.

As the introduction of technology in healthcare usually leads to changes in work organization and the roles of formal caregivers, the implementation of digital innovation should include the perspectives of users [27, 28]. Accordingly, the attitudes and experiences of the palliative care team involved in piloting the sensor system were explored in a qualitative study arm. To the best of our knowledge, there are no publications to date on the views of healthcare professionals in palliative care toward a contact-free sensor system [16]. In the following, we report on interviews with members of a multiprofessional palliative care team who were involved in a pilot study of a contact-free sensor system developed to detect and monitor distress in palliative care patients.

## Methods

### Objective, research questions, and design

To explore the attitudes and experiences of the palliative care team involved in piloting the sensor system, we pursued the following research questions: How do members of the palliative care team assess the benefits, limitations, and risks of a noninvasive, sensor system for the early detection of distress in patients who are unable to express themselves and its permanent use in the palliative care unit?

To gain insight into the broader context of attitudes towards digital health research, we wanted to know: How do they generally rate the use of research and health technologies in the field of end-of-life care?

Based on the constructivist paradigm [29], we chose a qualitative research design to explore as openly as possible the hitherto unknown views of healthcare professionals in palliative care concerning such a sensor system and to reconstruct their attitudes as well as subjective attributions of meaning and significance to the system.

### Setting

Between 2017 and 2019, we developed a contact-free, bed-bound sensor system (“iSenDi”) in a pilot study (Quickstart-Rev study) and tested it for six months in the palliative care ward (10 beds) of the University Medical Center Freiburg (Germany). The aim was to detect distress in patients who were no longer able to express themselves due to advanced illness and/or at the end of life. The multisensor system consisted of radar and piezo sensors, temperature and humidity sensors, and a microphone. The beds of patients who had provided proxy consent were equipped to record sensor data. At the same time, stress events (such as pain, shortness of breath or anxiety attacks) were documented by a study nurse who observed the patient noninvasively at night.

### Population and sampling

All the nursing staff, physicians, and psychosocial team members who were present on at least one shift when a measurement with the sensor system took place were considered potential interview partners. Members of the research team involved in the actual implementation of the sensor system were also approached for interviews.

A two-pronged sampling approach was chosen: (a) a purposive sampling strategy with the aim of maximizing variance in terms of different occupations, age, gender, work experience, and attitudes toward technological innovations at the end of life and (b) the selection and approach of respondents by the study physician as gatekeeper and collaborator.

### Recruitment

Recruitment took place in two phases (07–09/2020 and 01–02/2021). From the beginning of the study, all potential interview partners in the palliative care team were informed about the possibility of giving a voluntary interview at the end of the study. After being approached verbally or in writing by the study physician, they could decide whether to participate. All those who were approached participated in the interviews.

### Data collection

The semi-structured interviews were conducted face-to-face at the interviewees’ workplace. The interview guide was drawn up by a psychologist from the scientific evaluation team (COR) following a brainstorming session with the multiprofessional team (physician (CB), psychologist (COR), and psycho-oncologist (CaB)). The interview guide was piloted with one of the physicians and covered the following main topics: experiences with the sensor system, feasibility in everyday clinical practice, benefits and fears, and research and health technologies in the field of end-of-life care and patients who are no longer able to express themselves. The full interview guide can be found in Additional file 1.

In total, 20 interviews were conducted in two phases. Firstly, 14 interviews were carried out with members of each occupational group. After an initial phase of data analysis, we then primarily interviewed members of the psychosocial and medical teams (6 interviews) until the content was saturated.

All participants were interviewed by two psychologists from the scientific research team (COR and another psychologist Ulrike Viesel (UV)) who had several years of experience in qualitative research and interviewing. Some of the interviewers and team members knew each other as they belonged to the same department. All interviews were digitally audio-recorded and then transcribed verbatim. In addition, field notes were taken directly after the interviews.

### Data analysis

The interviews were analyzed within the framework of a method triangulation between October 2020 and March 2021. The main method used was content-structuring qualitative content analysis [30], which focuses on the development of a code system that maps the data material as a thematic matrix. In the first step, an intensive familiarization with the transcripts took place in accordance with the method; first individually, then in the scientific evaluation team, which consisted of a female social scientist (KS) with many years of experience in qualitative research and two female psychologists (UV and COR). The social scientist also knew some of the interviewees through several years of collaboration at the same department.

The texts were thoroughly read and discussed with regard to the research questions, and initial notes were made. This first step of qualitative content analysis was supplemented with the integrative basic method, a linguistic-descriptive analysis approach that leads to a data-centered interpretation by focusing on interaction between the interviewees, syntax, and semantics [31]. At the end of this first analysis phase, a case excerpt was created by UV and KS for 70% of the interviews, i.e. systematically organized initial case summaries [30].

In the second step, after all 20 interviews were available following the second data collection phase, thematic main and subcodes were developed by KS and UV with regard to the research questions, and these were described in a coding guide. In the third step, the two researchers each computer-coded half of the interviews.

The analysis was supported by MAXQDA software version 12. Specifically, the interviews were divided into sections of meaning and these text passages were then assigned to the respective codes or subcodes. A few new subcodes were formed inductively during this process through discourse between the two researchers. The coding was partially recoded by the other researcher, disagreements were discussed in team meetings or new coding was carried out. In the final step, the resulting main codes and subcodes were systematized and formulated by both researchers in terms of content and related to each other.

## Results

### Description of the sample

A total of 20 interviews were conducted with members of the multiprofessional palliative care team and the research team involved in the evaluation of the sensor system. The average duration of the interviews was 36 min (with the longest lasting 65 min and the shortest 15 min). A heterogeneous sample was obtained through purposive sampling (see Table 1).

**Table 1:** Description of the study sample

	Nurses (*n* = 8)	Physicians (*n* = 5)	Psychosocial team (*n* = 4)	Scientific team (*n* = 3)	Total (*n* = 20)
Gender					
Female	6	3	4	1	14
Male	2	2	0	2	6
Age					
20–30 years	0	0	1	0	1
30–40 years	3	3	2	1	9
40–50 years	3	1	0	2	6
50–60 years	2	1	1	0	4
Work/professional experience					
< 5 years	0	0	1	0	1
5–10 years	0	2	2	2	6
10–20 years	3	3	0	1	7
20–30 years	4	0	1	0	5
> 30 years	1	0	0	0	1

### Code system

The developed code system comprised the following main codes, which are reported below: perceived usefulness of the sensor system for current and future end-of-life care, perceived ease of use, limitations of the sensor system, fears and risks associated with the sensor system, evaluation of the routine use of the sensor system on the palliative care ward, and general attitudes toward health technologies and research at the end of life.

### Perceived usefulness for current and future end-of-life care

According to the interviewees, three target groups in particular could benefit from the sensor system: patients, nursing staff, and caregiving relatives. Due to the additional information channel and the improved monitoring situation, patients’ distress could be recognized earlier, suffering could be prevented, and patient safety (e.g. through fall prevention) could be increased, especially at night. Nursing staff also benefited from an increased sense of security with regard to restless patients and the knowledge that distress episodes would not go undetected.

*“I definitely see a benefit … because as a nurse you want to be able to recognize stress and then do something about it. But with patients like that [who can no longer express themselves*,* the authors]*,* that would actually mean that you have to be in the room around the clock and you can’t do that because you also have to look after other patients. That means that this time in between is always such a black hole for you and it leaves you with a bad feeling when you don’t know what’s happened. Maybe the person was suffering*,* and I just didn’t realize it.” (I01*,* nursing staff)*.

The same applied to relatives involved in care in the clinical setting: They could experience emotional and physical relief through additional monitoring.

In addition to the current care structures, the interviewees also discussed the future situation. Against the backdrop of demographic change and scarce personnel resources, gaps in personnel in the clinical and domestic context could also be filled by technology such as sensor systems. This would offer support and relief to nursing staff or family carers at home, allowing them to concentrate on their core areas. In the future, a sensor system could therefore be one building block among others for overcoming demographic challenges.

### Perceived ease of use

Initial technical challenges such as connecting the sensor system to the power line were reported, but the subsequent use of the sensor system was generally described as simple: beyond a mattress change, no other invasive measures were involved.

### Limitations of the sensor system

The limitations of the sensor system were primarily related to its capabilities and were assessed against the background of human care. Although the device was seen as useful for monitoring and alerting, it could not interpret the nature of the distress or decide what type of support or treatment should follow the stressful situation. The assessment of distress remained the task of the nursing staff, who described their expertise in this area as a mixture of patient observation, intuitive perception, and years of experience.

*“What can the device do? … No one here [patients on the palliative care ward*,* the authors] has no stress*,* do they? So*,* illness and especially dying is stress per se. And … we make sure that the nursing staff working here really have a lot of experience … to decide and recognize: Is this stress now in need of treatment … And that requires hearing*,* smelling*,* seeing and*,* above all*,* a lot of feeling*,* because that is simply at a level where … a lot of this holistic perception is needed to recognize whether it is tolerable or not*,* or whether it is bearable for the person or not. … but I don’t think that a device can help me with this*,* because I realize that this is simply only recognizable when I spend time with the person*,* i.e. what I experience in everyday life when I accompany someone who can no longer communicate this to me in words.” (I08*,* nursing staff)*.

Some of the interviewed nursing staff also expected little or no personal benefit in their own day-to-day work, as there was no need to use the alarm function for critical situations due to their own routine, experience, and intuition. For inexperienced colleagues, however, the sensor system was deemed rather helpful.

### Fears and risks associated with the sensor system

The potential risks raised by respondents included the fear that the sensor system could lead to rationalization and personnel savings/replacements.

*“I think my first thought was*,* oh*,* that’s blatant*,* … Basically*,* the idea here is to develop a technology that*,* if it works well*,* is ultimately suitable for replacing people and staff or somehow replacing this human care in the field of palliative care.” (I16*,* psychosocial team)*.

In addition, general technology risks, such as the production of artifacts, the loss of interpersonal connections or the uncontrollability of sensor systems in future settings, were anticipated. Some of the interviewees also saw risks with regard to data protection, such as data leaks, data trading or data misuse.

### Evaluation of the routine use of the sensor system on the palliative care ward

Future routine use was considered conceivable by some of the interviewees, but only under certain conditions by others: (a) if symptom burden and limited expressive capacity of the patient were given as important prerequisites and justification for the use of the sensor system, and (b) if it was structurally and procedurally ensured that providers and staff weighed up the risks and opportunities in terms of patient welfare before use and that staff acceptance was obtained: *“I think it depends very much on the institution which precautions*,* let’s say*,* need to be taken. I think it’s a decision that ultimately has to be made by the provider [of the institution*,* the authors] together with the staff. Is it more of a danger or is it a support? And how do we deal with it so that it’s really beneficial for the person who’s in bed?” (I02*,* nursing staff)*.

### General attitudes toward health technologies and research at the end of life

#### Role of advocate: openness to health technologies if beneficial for the patient

When asked about their attitudes toward health technologies and research at the end of life, some interviewees stressed that palliative care should be characterized by the limited use of technical devices and the simultaneous importance of holistic perception of the patient’s condition and interpersonal contact: *“high touch*,* low tech ... so rather less ... rather than more in the sense of no more monitoring(1) ... as a palliative care attitude” (I17*,* physician).*

In general, however, there was a positive and pragmatic attitude toward research and the use of health technology devices in palliative care if they promoted the well-being (or quality of life) of patients, e.g. by improving the quality of care. The use of technical and digital innovation and research were thereby always considered under the following aspects: (a) ethically responsible conditions (e.g. harmlessness, usefulness/meaningfulness, reasonableness/non-invasiveness at the end of life) and possible burdens for patients and (b) compatibility with professional nursing and/or palliative care attitudes and values (e.g. no restriction of holistic care through technical substitution/demanding research conditions).

In our study, this became clear with regard to the audio recording of the device and repositioning of the patients for study purposes. For some respondents, audio recording via the sensor system was highly invasive, as communication among nursing staff, patients, and relatives was experienced as disturbed or reduced (e.g. no mention of patient names in the context of the study situation due to data safety considerations). In addition, the recording of end-of-life conversations with relatives and the sounds of the dying situation were considered potentially problematic from an ethical point of view.

*“Well*,* it was at night*,* the recording function was running and the relatives were actually there to say goodbye … and this intimacy that should simply be there in this moment of dying … it was no longer there … really*,* for me the question is*,* when is such an intimate situation—… such a situation that is unique*,* it’s about one last time*,* it’s perhaps the last opportunity for the relatives to say goodbye*,* it’s the last hours … in a person’s life; when do you take away the special nature of this moment and the intimacy and this special dynamic—I find that highly questionable.” (I14*,* psychosocial team)*.

Other examples were the need to transfer seriously ill patients to a bed equipped with a sensor system and the inclusion of a dying patient and the resulting influence on the terminal situation, which were also critically assessed. These instances were perceived by some nurses as a serious breach of standards in the palliative care unit (e.g. the rigorous assessment of end-of-life transfers, which was perceived as not being adhered to in favor of study inclusion).

Thus, in the interviews, the team took on a protective or advocacy role toward the patients by demanding an individual, ethically responsible, and situation-specific use of innovation and research: *“I can see that studies and research are also important*,* can’t I? But I also realize that I see myself first and foremost as an advocate and representative of the person lying in the bed. And I think and speak and make decisions entirely from the patient’s perspective.” (I08*,* nursing staff)*.

### Differences in openness

In addition, the overall assessment showed a continuum in terms of openness to technical innovations and research with two opposing perspectives:

- Some interviewees wanted as little technology as possible at the patient–carer micro level and supported research that is meaningful for the patients involved: *“So*,* for people with serious illnesses*,* especially in the terminal stage*,* I actually prefer as little technology as possible*,* only what is helpful.” (I18*,* nursing staff)*.

b) Other respondents at the meso level supported opening up palliative care to technology, innovation, and research: *“When I received the information about the study and was told that it was starting soon*,* I was pleased that we are also very active in this area and that we are testing an interesting approach and*,* yes*,* I would say that we are also a bit innovative here in palliative care.” (I06*,* physician)*.

The representatives of both perspectives used arguments based on the well-being of patients: some, especially in the nursing profession, were concerned with the well-being of current patients, while others, especially in the medical profession, felt that supporting new developments in the field of palliative care would improve the possible well-being of future patients.

Accordingly, some of the physicians tended to perceive the nursing staff’s attitude as challenging when it came to conducting innovative studies: *“Well*,* we live in a culture of cooperation here*,* where everyone is allowed to say ‘no’*,* which is the right thing to do. But that also makes research more difficult*,* because then I always have to do a lot of persuading*,* whereas in other areas of medicine I could simply say—from a position of*,* let’s say*,* medical authority—this is what we’re doing now and then the nursing staff wouldn’t say anything against it. I don’t want to say that this is generally desirable*,* because of course a lot comes from a critical attitude*,* but … it makes … some research projects more difficult … in some cases*,* I wouldn’t even go in certain directions or consider them because I know that I wouldn’t be able to push it through in the team.” (I10*,* physician)*.

In the case of the sensor system, the nursing staff shared the physicians’ view of their sometimes-hesitant attitude when recruiting: *“In principle*,* I think this study is very important*,* we all think it’s important and the system is also very important in principle … but when I’m at work*,* that’s where my focus is and everything else is totally secondary … I personally always proceed according to the principle: Not more than necessary … and to do as much as necessary for a patient*,* especially among palliative care patients—in this respect*,* I always found it an internal hurdle to bring in patients or even suggest them.” (I11*,* nursing staff)*.

Occasionally, however, they also referred to a lack of involvement in the recruitment process or the feeling of being left out: *“If you are screening study participants*,* then perhaps those … closely involved should also be brought on board to see whether that makes sense? … so*,* we nurses are the ones who have to implement this*,* so we experience the stress [of the patients*,* the authors]*,* we experience the outcome of therapies*,* and we are the ones who are given information under the table that is not mentioned during the ward round. That means we get the stress very directly and more unfiltered than some others in the team”. (I11*,* nursing staff).*

## Discussion

In this qualitative study, we explored the attitudes of a multiprofessional palliative care team toward the prototype of a sensor system for the early detection of distress in patients with limited means of communication. The results show the anticipated usefulness for patients, nursing staff, and caregiving relatives, but also its limitations, fears regarding the sensor system and the protective role of health professionals towards patients with regard to the introduction of health technology and research.

The results also suggest that the multiprofessional field of palliative care is subject to special conditions with regard to researching health technologies and their acceptance. As in other areas, e.g. aging [32] or home care [33], research into the acceptance of technology in end-of-life care should be expanded to include context-specific factors, such as a strong focus on multiprofessional and interdisciplinary work. As our study shows, this involves both profession-specific values and interdisciplinary challenges in the palliative context. This should always be kept in mind when planning and conducting research on technological innovations.

### Impact on professional values

- Focus on patient well-being.

In line with the general orientation of palliative care [34, 35], the interviews show that the promotion of quality of life and the alleviation or prevention of suffering are deemed benchmarks for the introduction of health technologies and accompanying research.

In terms of research ethics, there was a particular sensitivity among the interviewees with regard to the question of whether such innovations and research that specifically concern the end-of-life phase may jeopardize values of patient well-being. As has been shown in other studies [36, 37], the interviewees assumed an advocate position for the palliative care patient as part of their professional role —in the case of the present study, this was further reinforced by the fact that the patients could no longer communicate and the associated greater dependency.

Even if it is undisputed that the risks and benefits of the sensor system had to be assessed individually for each patient in the present study, it is also necessary to consider when advocacy turns into overprotective gatekeeping and thus hinders technical innovations and research. In addition, the fundamental willingness of palliative care patients to participate in studies [38–40] might be neglected in team assessments. Focusing primarily on the vulnerability aspects and, thus, disregarding the patient’s potential agency, might also constitute a form of discrimination.

b) Impact on the professional understanding of nursing.

In accordance with other studies, the results show that technical-digital innovations and the associated research can question values related to the professional understanding of nursing and holistic care [41–45]. The nursing staff did not see themselves threatened by the sensor system, as their core competence of inference, i.e. drawing conclusions about what action to take following a stress assessment, was not replaced [46]. However, it is also clear from the analysis that, for example, restrictions in communication due to the audio-recording feature of the sensor system were experienced as a break with the desired care practice at the end of life. In addition, future technical-digital innovation was associated with the fact that it could partially replace human perception and assessment of the patient’s condition, thereby influencing working conditions as well as professional practice (especially care) at the end of life. This aspect has also been expressed as a general concern in the palliative discourse beyond the scope of this study [47]. While the use of technology for patients in the palliative care unit can currently be considered with a focus on patient well-being, and the rapid development of artificial intelligence is likely to replace audio recording with real-time, memoryless analysis, the consequences of technology use on the profession and general working conditions cannot be directly controlled.

### Interprofessional differences with regard to innovation and research

Our study also revealed interprofessional differences in opinions on the subject of innovative research in the palliative care setting. One example stemming from our results is the inclusion of a dying patient, which was initiated by physicians. Although decided collectively by the team, the actual transfer of the dying patient to the bed equipped with the sensor system was experienced negatively by the nurses carrying out the procedure. In other words, there was a breach between the medically initiated study inclusion and the experienced professional nursing responsibilities in practice. This goes hand in hand with the feeling that the otherwise flat hierarchy and the participation of all professions in decision-making were sometimes ignored during the recruitment process, i.e. the nursing perspective was not adequately heard. The physicians’ perspective, on the other hand, indicates that at times, the nursing staff could make innovative research and specifically the implementation of this study more difficult.

The analysis of the interview data revealed that the right to veto can be used not only to protect patients but also to introduce and assert one’s own professional principles in the multiprofessional dialog. On the nursing side, the focus of research projects is primarily on current patients and their quality of life or on everyday nursing care, including its mission to provide good patient care and promote quality of life. From the medical perspective, the strategic, future-oriented view, which targets the well-being of future patients as a whole through innovation and research, is also important. In summary, interprofessional collaboration is a basic requirement and a particular strength of palliative practice [48]. On the other hand, the well-known barriers such as “problematic power dynamics, poor communication patterns, lack of understanding of one’s own and others’ roles and responsibilities and conflicts due to varied approaches to patient care” ( [49], p.3) are also evident in our findings.

### Practical implications

Our study is consistent with other empirical findings: The involvement of health staff in the development and implementation of technology is central to its acceptability [50–52]. Future research into technical-digital innovation in the palliative care field should therefore always take into account the professional values and attitudes of healthcare staff as important stakeholders. A breach of these values can lead to the failure of an implementation, not because of the technical-digital device itself and its benefits but due to unforeseen, context-dependent shared beliefs and norms that counteract implementation. Context-related analyses, such as those used in implementation research [53], could be helpful here.

Particularly with regard to procedural justice, researchers should clearly encourage the healthcare professionals involved to contribute their own professional perspectives and concerns. An adequate design of the communication process during introduction and implementation is an important criterion, which should also include the identification of participation and codesign [54]. The team’s concerns during study implementation should also be documented as part of a formative evaluation. In this way, critical incidents such as the repositioning of a dying patient can be addressed promptly and dealt with by the team.

### Fields of implementation

Successful implementation of sensor systems in palliative care might in future facilitate the detection of distress events in patients that are unable to call for help for various reasons. These could be very frail patients at the end of life, but also patients with cognitive impairment due to delirium or dementia [55]. Accordingly, applications outside the palliative care ward in nursing homes are also conceivable [56].

Furthermore, in the scenario of a future pandemic and resulting dying in isolation, as was the case during the COVID-19 pandemic [57], the use of sensor systems to detect distress would potentially be of great benefit.

### Strengths and limitations

The strengths of the study clearly lie in its qualitative approach, which made it possible to gain deeper insight into the attitude level and the respective constructions of meaning. In addition to manifest content, it was also possible to identify latent meaning such as impact on professional values and professional self-image.

Only one team at one location was interviewed; other structures may allow further attributions of meaning to become visible. Furthermore, due to challenges with the development and testing of the sensor system, only three patients were included. While experience with more patients could have led to additional insights, we want to point out that the discussions in the multidisciplinary team regarding the implications of the sensor systems were extensive, including good clinical practice training of the study nurses who were part of the nursing staff.

We had also planned to correlate the stress events recorded by the nurses with the sensor data to potentially develop an algorithm to predict distress episodes. However, the sensor system had not yet reached market readiness.

At the time the study was conducted, there was no nursing scientist in the scientific team who could have been involved in the development of the interview guideline or the analysis. This would have strengthened the multi-professional orientation of the study. Finally, it would have been beneficial to also explore relatives’ views to obtain a systemic perspective; this was not possible due to the small number of participants.

## Conclusions

Digital and technical innovations, such as the sensor system we have introduced, might play a key role in ensuring that palliative care can continue to be provided under appropriate conditions in the future. As palliative care professionals integrate digital health innovations into care, their involvement is important from the outset of development and implementation. Our data also show that one should not assume a stereotypical rejection of technology, innovation, and related research in the field of palliative care, but rather a differentiated picture of a general openness under specific conditions that go hand in hand with a holistic understanding of care and the observance of basic palliative attitudes.

### Electronic supplementary material

Below is the link to the electronic supplementary material.

Supplementary Material 1. Additional file 1: Interview guide of the study.

## Data Availability

The interview transcripts cannot be shared as a whole, as there is no consent from the participants. The code system and extracts of the coding are available from the corresponding author Katharina Seibel (KS) on reasonable request.
