# Supplementary material for: Implementation of a digital distress detection system in palliative care: qualitative data on perspectives of a multiprofessional palliative care team
Source: BMC Palliat Care. 2024 Aug 7;23:203. doi: 10.1186/s12904-024-01530-3 (PMC11304939; doi:10.1186/s12904-024-01530-3)
Supplement: Supplementary file 1 — Supplementary Material 1. Additional file 1: Interview guide of the study. [file 12904_2024_1530_MOESM1_ESM.docx]

**Additional file 1 - INTERVIEW GUIDE**

| **1) Main question**  The measurements with the multi-sensor device and the observations made by the study nurses continued until last fall. I would now like to ask you to tell me about your experience of the study. I am interested in any details that seem important to you. Please take your time, I will not interrupt you for the time being. | **Possible follow-up questions**   - What went through your mind when you first heard about the study? - Did you have any concerns before starting the study? / What were they? - What are the positive / negative aspects of the project for you? - Was there anything that surprised you? |
| --- | --- |
| **2) Main question**  To what extent have you had to deal with the device in your day-to-day work? Can you describe this in more detail? | **Possible follow-up questions**   - During patient observation, the background sounds in the patient's room was also recorded with a microphone. How did you experience the recording function of the device? - Do you have any concerns about the recording function? - And how do you feel about the presence of the study nurse in the patient's room? - How did you experience the reactions of the relatives of the participating patients? - In your opinion, has the study had a general impact on the team? - What would you say? Do you see any benefit in using the device on the palliative care ward? |
| **3) Main question**  How do you generally assess the use of health technologies in the care of people with a serious illness? | **Possible follow-up question**   - Is there anything that should be considered regarding the use of technical support systems in the care of seriously ill people? |
| **4) Main question**  What are your general thoughts on research conducted on seriously ill patients? | **Possible follow-up questions**   - In your opinion, under what conditions should research be conducted on palliative care patients? - And especially in the case of patients who are unable to make decisions: How do you feel about these patients being included in studies? - How do you feel about relatives making the decision to participate in the study? - What problems or risks do you see in this? |
| **5) Concluding the interview**  Is there anything else you would like to say that has not yet come up in our interview? |  |
| **Thank you very much for the interview!** | |
